# Supplementary material for: Identification and characterization of vasoactive intestinal peptide receptor antagonists with high-affinity and potent anti-leukemia activity
Source: J Biol Chem. 2025 Dec 30;302(3):111127. doi: 10.1016/j.jbc.2025.111127 (PMC13080581; doi:10.1016/j.jbc.2025.111127)

**Supplementary Figure 1**

A. VIP & VPAC1 B. ANT308 & VPAC1


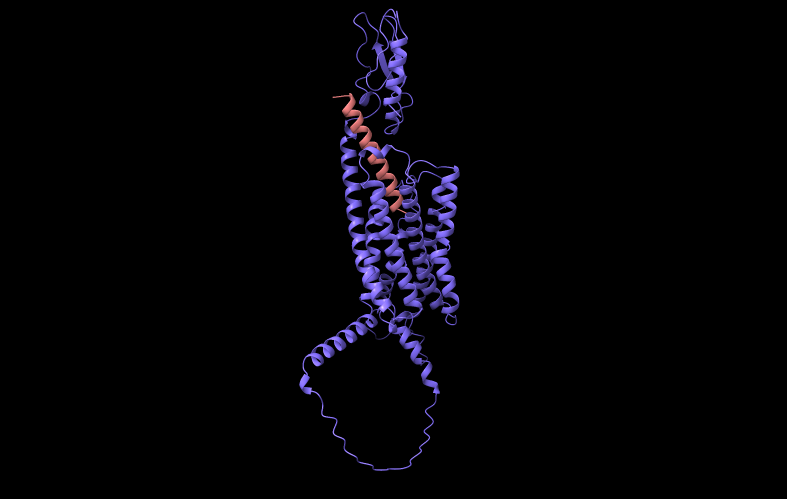

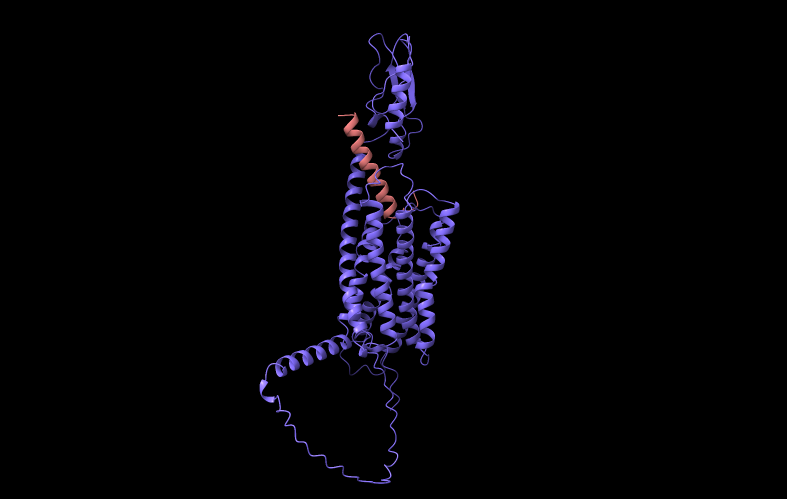


**Supplementary Figure 1. Human VIP-VPAC1, ANT308-VPAC1 complex solved by Alphafold.** Secondary structure comparison of human VPAC1 (purple multi-domain complex, full structure including transmembrane & intracellular domains) binding to VIP (pink peptide helices and beta sheets) or ANT308 (pink peptide helices and beta sheets).

**Supplementary Figure 2**

**
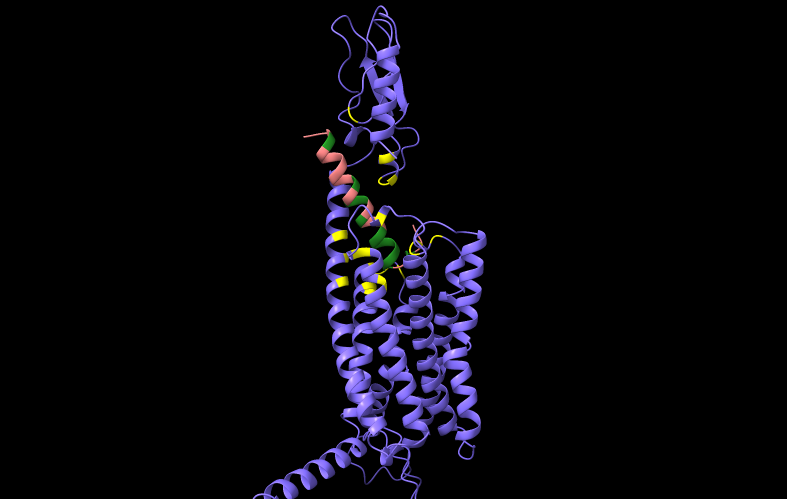

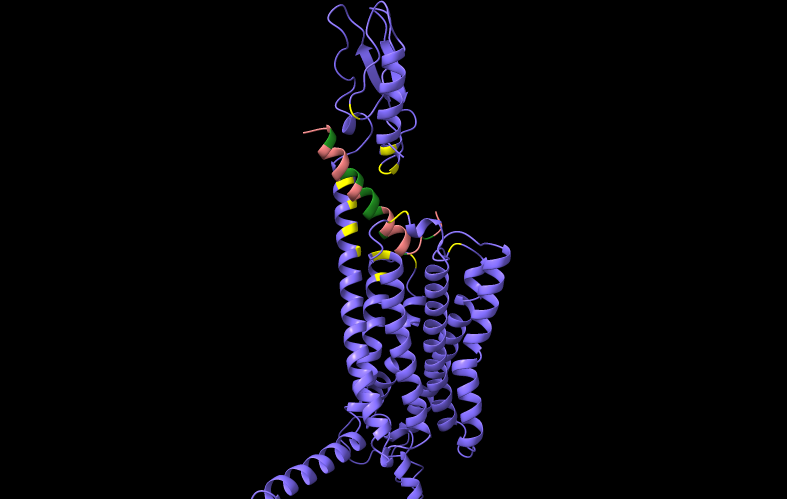
**A. ANT300 & VPAC1 B. ANT008 & VPAC1


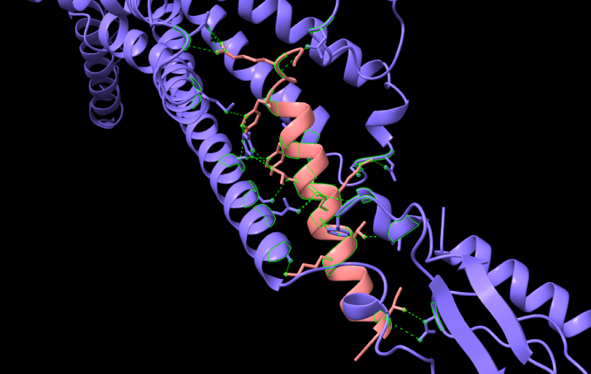

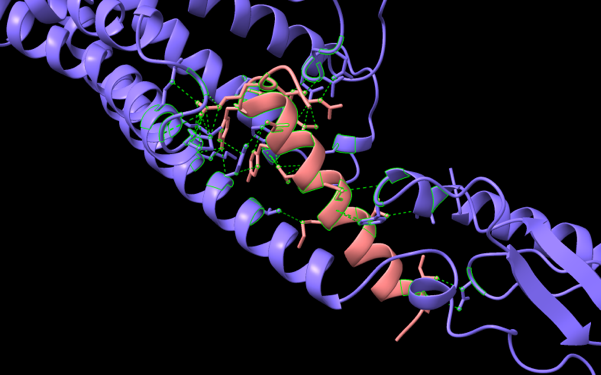
C. ANT300 & VPAC1 Zoom-In D. ANT008 & VPAC1 Zoom-In

**Supplementary Figure 2. Human ANT300-VPAC1, ANT008-VPAC1 complex represented by Alphafold.** Secondary structure comparison of human VPAC1 binding to ANT300, ANT008. Human VPAC1 (purple multi-domain complex, VIP binding interfaces highlighted in yellow). (*A)* ANT300, (*B)* ANT008 (pink peptide helices and beta sheets, VPAC1 binding interfaces highlighted in green). (*C-D)* Zoom-In view of ANT300 or ANT008 binding pockets at the transmembrane bundle of the VPAC1 receptor, pseudo-bonds are highlighted in green.

**Supplementary Figure 3**

A. VIP & VPAC1 B. VIPhyb & VPAC1 C. ANT308 & VPAC1


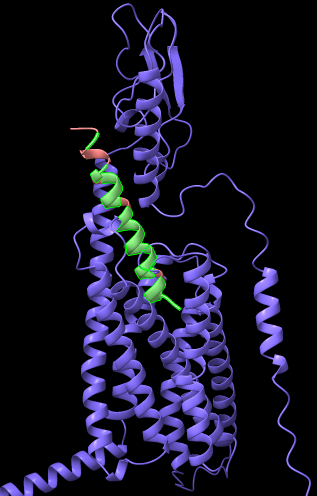

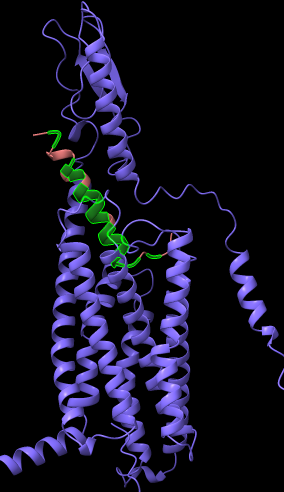

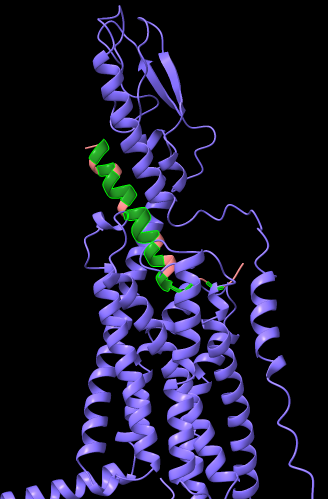


D. VIP & VPAC2 E. VIPhyb & VPAC2 F. ANT308 & VPAC2


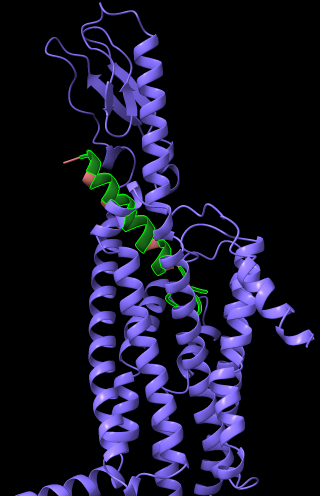

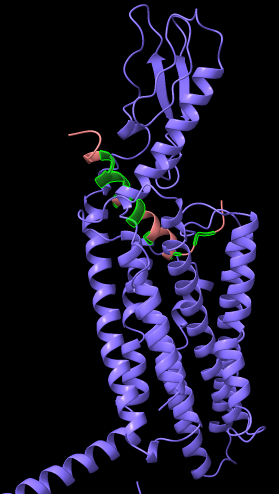

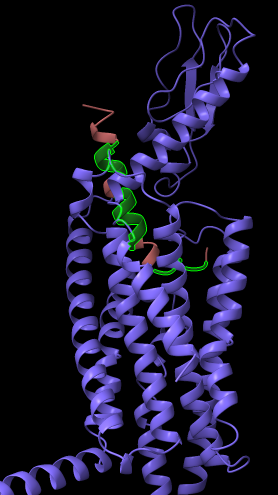


**Supplementary Figure 3. The binding of peptide antagonists to mouse VPAC1, VPAC2 as represented by Alphafold.** Secondary structure comparison of mouse VPAC1 binding to VIP, ANT308, and VIPhyb. Mouse VPAC1 (purple multi-domain complex). *(A)* VIP, *(B)* VIPhyb, *(C)* ANT308 (pink peptide helices and beta sheets, VPAC1 binding interfaces highlighted in green). *(D-F)* Mouse VIP-VPAC2, VIPhyb-VPAC2, ANT308-VPAC2 complex solved by AlphaFold. Secondary structure comparison of mouse VPAC2 binding to VIP, VIPhyb, and ANT308. Mouse VPAC2 (purple multi-domain complex). *(D)* VIP, *(E)* VIPhyb, *(F)* ANT308 (pink peptide helices and beta sheets, VPAC2 binding interfaces highlighted in green).

**Supplementary Figure 4**

**
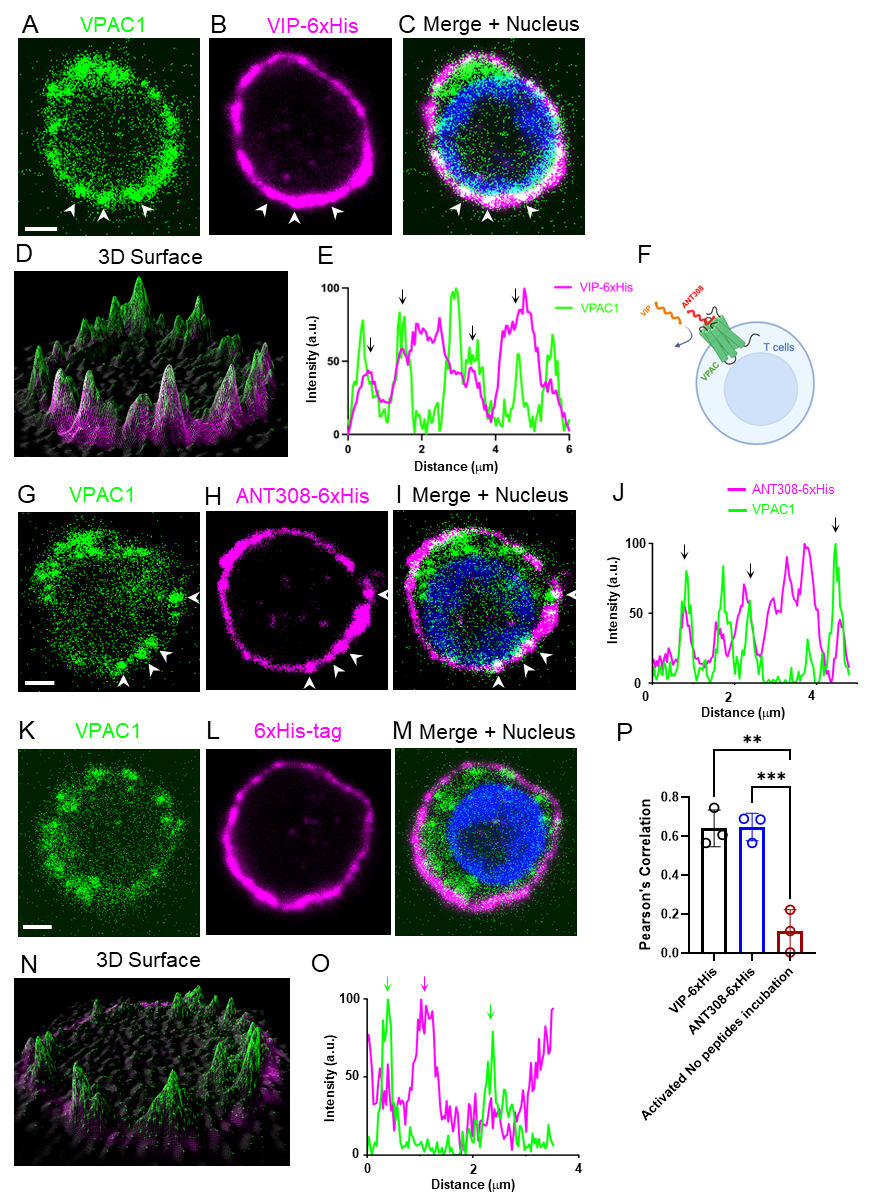
**

No peptide

anti-His Ab

**Supplementary Figure 4. Immunostaining showing VIP-6xHis & ANT308-6xHis colocalize with VPAC1 on human T cells surface**. Isolated & pooled T cells from 2 healthy donors were incubated with *(A-E)* 5μM VIP-6xHis, *(G-J)* ANT308-6xHis, *(K-O)* no peptide in the presence of soluble human CD3/CD28/CD2 T cell activator & human IL-2 (50 IU/ml) on Poly-L-glycine coated coverslip for 24hr, fixed with 4% Paraformaldehyde and stained with VPAC1 (green), 6x-His (magenta) & Hoechst (blue). *(A-C)* *en face* view, *(D)* 3D view, *(E)* line scan profiles (black arrows) showing VIP-6xHis colocalizes with VPAC1 on T cells; *(F)* schematic representation showing VIP-6xHis & ANT308-6xHis binding to VPAC1 on T cells. *(G-I)* *en face* view, *(J)* line scan profiles (black arrows) showing ANT308-6xHis colocalizes with VPAC1 on T cells. *(K-M)* *en face* view, *(N)* 3D view, *(O)* line scan profiles (green, magenta arrows) showing 6x-His signal does not overlap with VPAC1 signals on activated T cells in the absence of a 6x-His tagged peptide. (*P*) Quantification of co-localization using Pearson’s correlation coefficient (R-value). Data are shown as mean ± SD with individual data points (n=10). Statistical significance was determined by one-way Anova with post-hoc multiple comparisons. **** p < 0.0001, *** p < 0.001. Scale 2.5µm.

**Supplementary Figure 5**

C

D

A

B


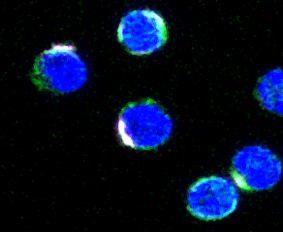

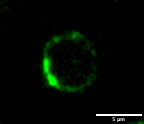

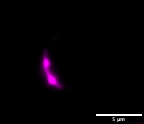

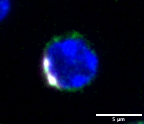


**WT T-cells**

**Merge + Hoechst**

**ANT308-6xHis**

**VPAC 1**


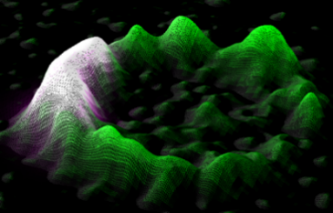


F

E

J

I

H

G


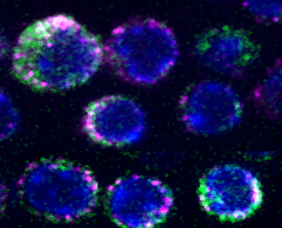

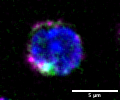

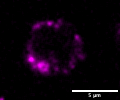

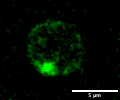


**WT T-cells**

**Merge + Hoechst**

**VIP-6xHis**

**VPAC 1**

Merge + Nucleus

L

K


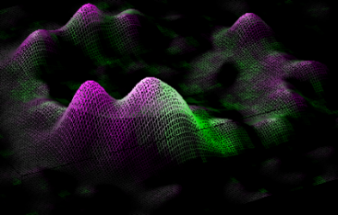


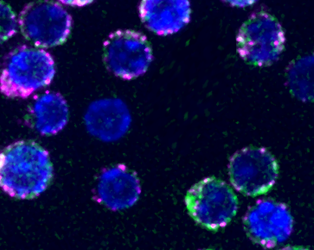

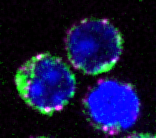


P

O

N

M

**Merge + Hoechst**

**VIP-SCRAM-6xHis**

**VPAC1**


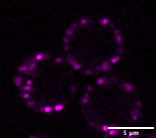

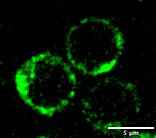


**WT T-cells**

S

R

Q


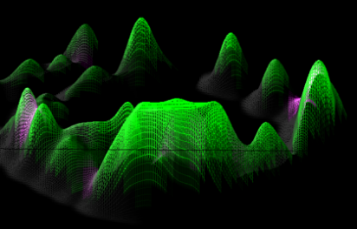


**Supplementary Figure 5**. **Immunostaining showing 6xHis-ANT308, 6xHis-VIP, and 6xHis-Scrambled-sequence peptides colocalization with VPAC1 on murine T-cells surface.** Isolated T-cells from naïve wild-type (WT) C57BL/6 mice were activated with α-CD3/α-CD28 beads and incubated with 10µM (A-F) ANT308-6xHis, (G-L) VIP-6xHis and (M-R) VIP fully SCRAM-6xHis in the presence of IL-2 (30 IU/mL) for 1 hour. The cells were fixed with 4% Paraformaldehyde, stained with VPAC1 (green), 6xHis-tag (magenta) & Hoechst (blue) and visualized on Cell-Tak coated glass-bottom plates. (A-C, G-I and M-O) *en face* view, (D,J & P) representative field view, (E, K & Q) 3D view, (F, L & R) line scan profiles showing colocalization patterns of ANT308-6xHis, VIP-6xHis and VIP-fully scrambled-6xHis with VPAC1 on T-cell membrane. Relative fluorescence intensity (RFI), arbitrary unit. (S) Quantification of co-localization using Pearson’s correlation coefficient (R-value). Data are shown as mean ± SD with individual data points (n=10). Statistical significance was determined by one-way Anova with post-hoc multiple comparisons. **** p < 0.0001, *** p < 0.001. Scale 2.5µm.

Merge + Nucleus

**Supplementary Figure 6**


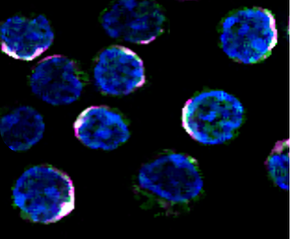

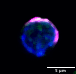

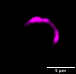

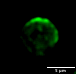


***Vipr2* KO T-cells**

D

**Merge + Nucleus**

C

**ANT308-6xHis**

B

A

**VPAC 1**

E

I

H

G

F


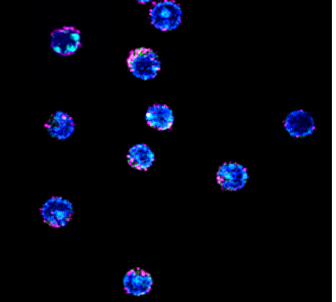

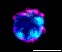

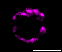

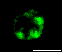


**VPAC 1**

**Scramble-6xHis**

**Merge + Nucleus**

***Vipr2***

**KO T-cells**

J

K

**
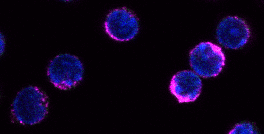
**
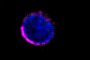

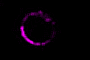

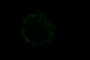


***Vipr1* KO T-cells**

O

Nc

M

L

**Merge + Nucleus**

**ANT308-6xHis**

**VPAC 1 Control**

P

**Supplementary Figure 6. ANT308-6xHis co-localizes with VPAC1 in *Vipr2 KO* murine T-cells.** (A–H) *Vipr2* knockout (KO) T-cells stained for VPAC1 (green) and either (A-E) ANT308-6xHis peptide (magenta) or (F-J) Scramble-6xHis control peptide. Strong co-localization of ANT308 with VPAC1 is observed in *Vipr2* KO, whereas the His-tagged VIP-scrambled-sequence peptide shows no overlap. (A-C, F-H and K-M) *en face* view, (D, I & N) representative field view, (E & J) line scan profiles. (L–P) *Vipr1* knockout (KO) T-cells stained for VPAC1 (green) show no detectable VPAC1 signal, consistent with the *Vipr1* knockout genotype. ANT308 binding remains detectable at the cell membrane, suggesting VPAC1-independent binding, likely to VPAC2. (K) Quantification of co-localization using Pearson’s correlation coefficient (R-value). Data are shown as mean ± SD with individual data points (n=10). Statistical significance was determined by one-way Anova with post-hoc multiple comparisons. **** p < 0.0001. Scale 2.5µm.

**Supplementary Figure 7**

A B C D

**
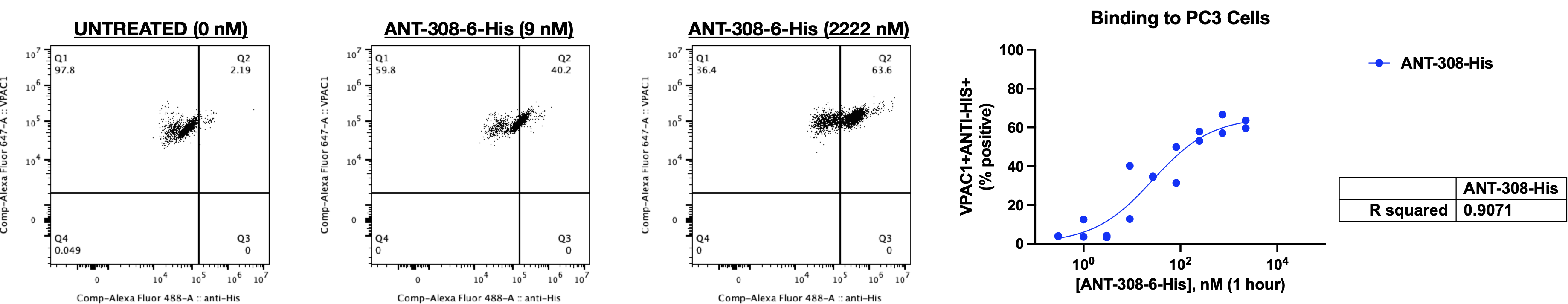
**

**Supplementary Figure 7. Flow cytometry analysis shows a dose-dependent increase in ANT308- VPAC1 double positive PC3 cells.** PC3 cells incubated with A) 0 nM, B) 9 nM ANT308-6xHis, or C) 2222 nM ANT308-6xHis for one hour, stained with saturating concentrations of Alexa Fluor 488-anti-His and Alexa Fluor 647-antiVPAC1 antibodies, washed, and analyzed by flow cytometry. D) Percentage of ANT308-6xHis-VPAC1 double positive cells with a range of ANT308-6xHis concentrations.

**Supplementary Table 1**

| VIPhyb | Mouse VPAC1 | ANT308 | Mouse VPAC1 |
| --- | --- | --- | --- |
| *K1* |  | ***K1*** |  |
| *P2* |  | ***P2*** |  |
| *R3* | D364, K371 | ***R3*** | D364, K371 |
| *R4* |  | ***R4*** |  |
| *P5* | I290 | ***P5*** | I290 |
| *Y6* | Y140, M372, L376 | ***Y6*** | Y140, M372, L376 |
| T7 | K196 | T7 | K196 |
| D8 | I290, T289 | ***S8*** |  |
| N9 | Y140 | ***D9*** |  |
| Y10 | Y140, D141, F201, | Y10 | Y140, D141, F201 |
| T11 | D288 | T11 | D288 |
| R12 |  | R12 |  |
| L13 | Y140 | L13 | Y140 |
| R14 | L200, F201 | R14 | L200, H208, F201, N203 |
| K15 | E36 | K15 | P33 |
| Q16 | L92, F93 | Q16 | L92 |
| M17 | T137 | M17 | E133 |
| A18 | H208 | A18 |  |
| V19 |  | V19 | F93 |
| K20 | E133, F93 | K20 | E133, F93 |
| K21 |  | K21 | E205 |
| Y22 | L40 | Y22 | L40 |
| L23 |  | L23 |  |
| N24 |  | N24 | H119 |
| S25 |  | ***L25*** |  |
| I26 | N69 | I26 | N69 |
| L27 | Y118, L70 | L27 | Y118 |
| N28 |  | N28 |  |

**Supplementary Table 1. Amino acid residues involved in Mouse VPAC1 and peptide interactions predicted by ChimeraX.** Differences from VIP sequence are indicated by ***bold, underlined, and italicized*** letters. Residue numbers were counted directly within the Mouse *Vipr1* sequence (UniProt, P97751).

**Supplementary Table 2**

| VIPhyb | Mouse VPAC2 | ANT308 | Mouse VPAC2 |
| --- | --- | --- | --- |
| *K1* |  | ***K1*** |  |
| *P2* |  | ***P2*** | W280 |
| *R3* | P347 | ***R3*** | P347, I350 |
| *R4* | D275 | ***R4*** | D275 |
| *P5* |  | ***P5*** | W280 |
| *Y6* | Q355, E359 | ***Y6*** | Q355, I356 |
| T7 |  | T7 |  |
| D8 |  | ***S8*** |  |
| N9 |  | ***D9*** |  |
| Y10 | Y129 | Y10 | K126, Y183 |
| T11 |  | T11 | D272, Y183 |
| R12 |  | R12 | N80 |
| L13 |  | L13 | Y122 |
| R14 | D179, Y183, Y122 | R14 | Y183, K126 |
| K15 | D179, D272, T273, F81 | K15 | F81, R25 |
| Q16 | F81 | Q16 | F81, P113, Y110 |
| M17 | K118, I119, Y122 | M17 |  |
| A18 |  | A18 |  |
| V19 | F78, F81, F26 | V19 | F26, F78 |
| K20 | D115 | K20 | D112, Y110 |
| K21 | E23 | K21 | E23 |
| Y22 | I30, H27 | Y22 | I30, N57, F26, H27 |
| L23 | Y110, F78 | L23 |  |
| N24 |  | N24 |  |
| S25 |  | ***L25*** |  |
| I26 |  | I26 |  |
| L27 |  | L27 |  |
| N28 |  | N28 |  |

**Supplementary Table 2. Amino acid residues involved in Mouse VPAC2 and peptide interactions predicted by ChimeraX.** Differences from VIP sequence are indicated by ***bold, underlined, and italicized*** letters. Residue numbers were counted directly within the Mouse *Vipr2* sequence (UniProt, P41588).

**Supplementary Table 3**

| Peptide | Predicted Binding affinity surrogate scores to VPAC1 | iPTM |
| --- | --- | --- |
| ANT308-6xHis | 14.25 | 0.79 |
| ANT308 | 12.41 | 0.85 |
| VIP-6xHis | 12.99 | 0.82 |
| VIP | 11.02 | 0.89 |

**Supplementary Table 3. AlphaFold3 predicted similar binding-affinity surrogate scores for VIP & ANT308 with or without a C-terminal 6xHis tag.** Binding affinity surrogate scores: lower scores imply better receptor binding. ipTM is a modeling confidence score that measures the accuracy of the predicted relative positions of the subunits forming the protein-protein complex. Values above 0.8 represent predictions with high precision, while values below 0.6 suggest a low probability of binding accuracy.

**Antibodies for Western Blots**

| Target | Origin | Reference | Catalog # | Concentration |
| --- | --- | --- | --- | --- |
| Phospho-CREB (Ser133) | Rabbit monoclonal | Cell Signaling Technology | 9198 | 1 : 1000 |
| CREB | Rabbit monoclonal | Cell Signaling Technology | 9197 | 1 : 1000 |
| β-actin | Mouse monoclonal | Cell Signaling Technology | 3700 | 1 : 1000 |

**Antibodies for Flow Cytometry**

| Target | Clone | Fluorochrome | Supplier | Reactivity | Catalog# | Dilution |
| --- | --- | --- | --- | --- | --- | --- |
| CD3 | UCHT1 | PE-CF594 | BD Biosciences | Human | 562310 | 1 to 100 |
| CD8 | SK1 | FITC | BioLegend | Human | 344704 | 1 to 100 |
| CD4 | RPA-T4 | APC-Cy7 | BioLegend | Human | 300518 | 1 to 100 |
| CD69 | FN50 | PE-Cy7 | BioLegend | Human | 310912 | 1 to 100 |
| Perforin | dG9 | Alexa Fluor 647 | BioLegend | Human | 308110 | 1 to 100 |
| Granzyme B | GB11 | Pacific Blue | BioLegend | Human | 515408 | 1 to 100 |
| Ki67 | Ki-67 | BV605 | BioLegend | Human | 350522 | 1 to 100 |
| VIP | OT15B5 | PE | OriGene | Human, Mouse | CF806852 | 1:50 |
| Mouse IgG1 | MOPC-21 | PE | BD Parmingen | Mouse | 555749 | 1 to 100 |

**Mass Spectrometry**

*ANT308*


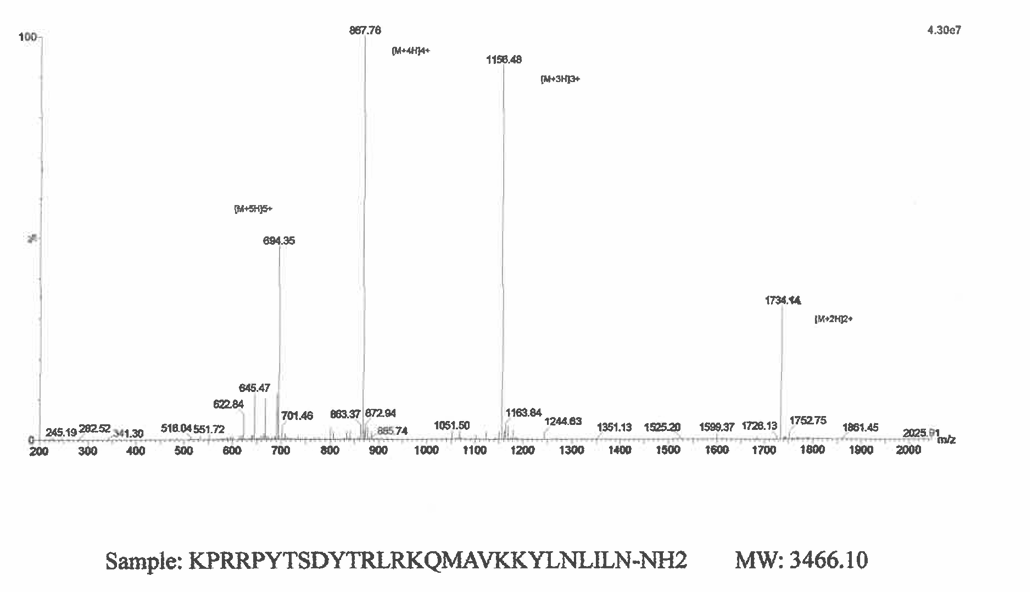


*ANT058*


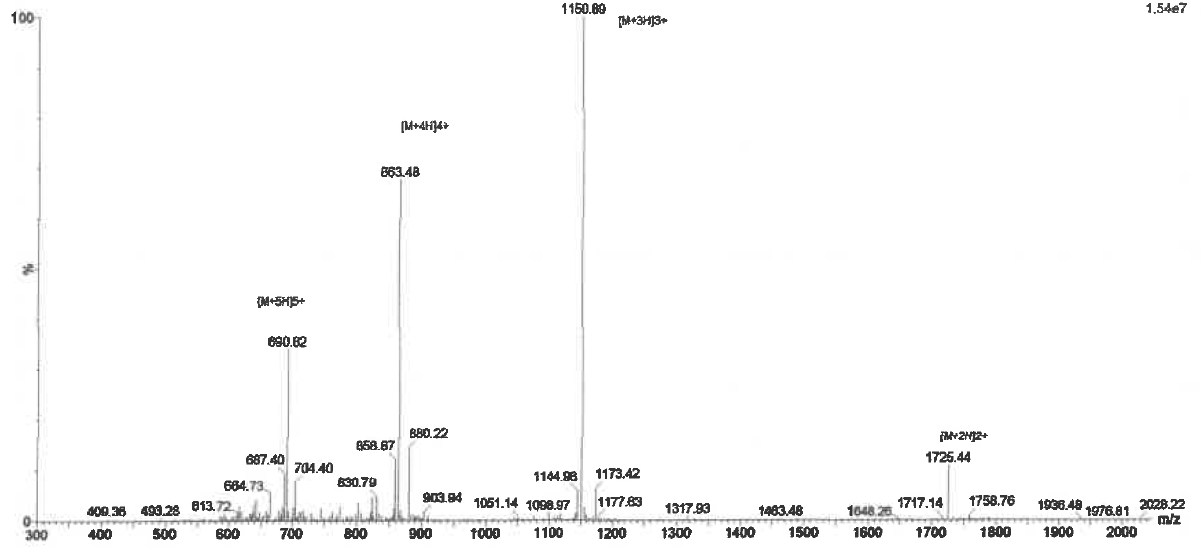


*ANT107*


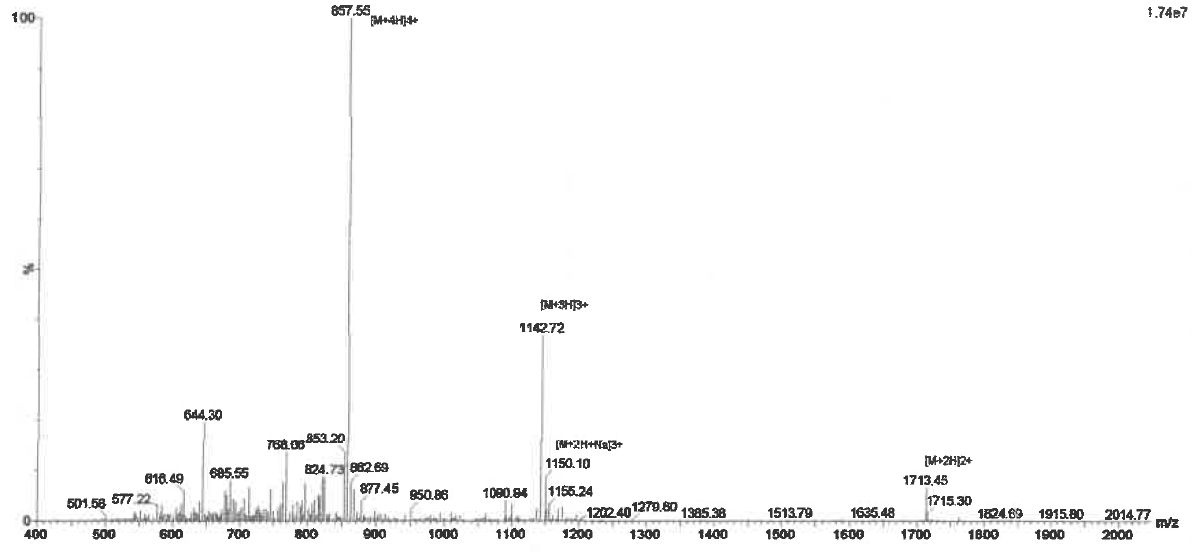


**HPLC**

*ANT308*

**
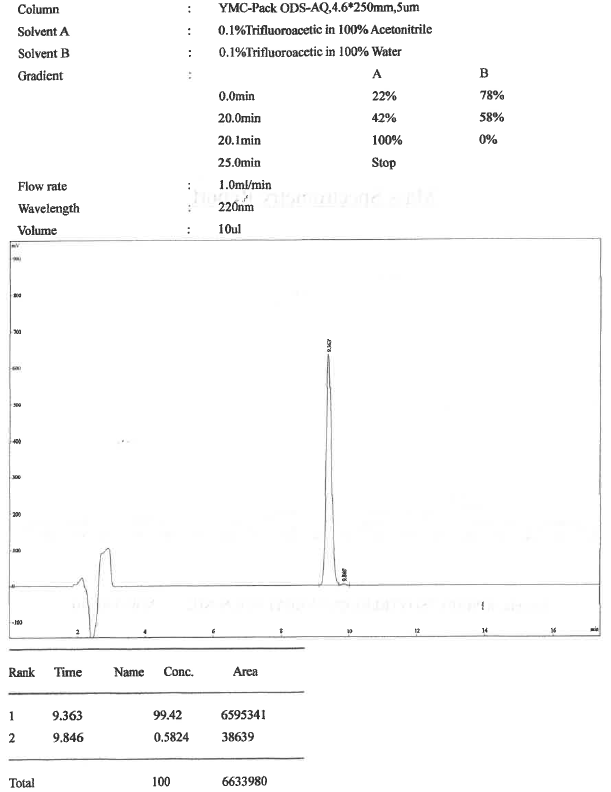
**

*ANT107*


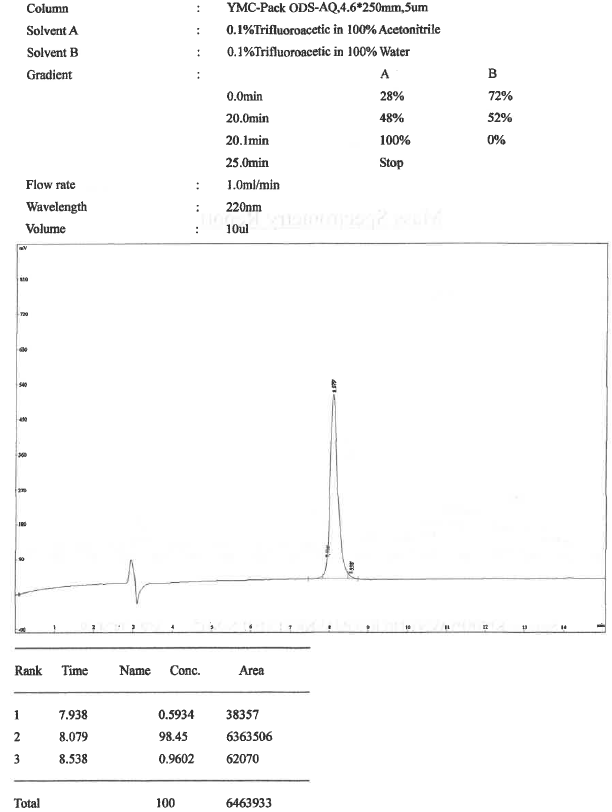


*ANT114*


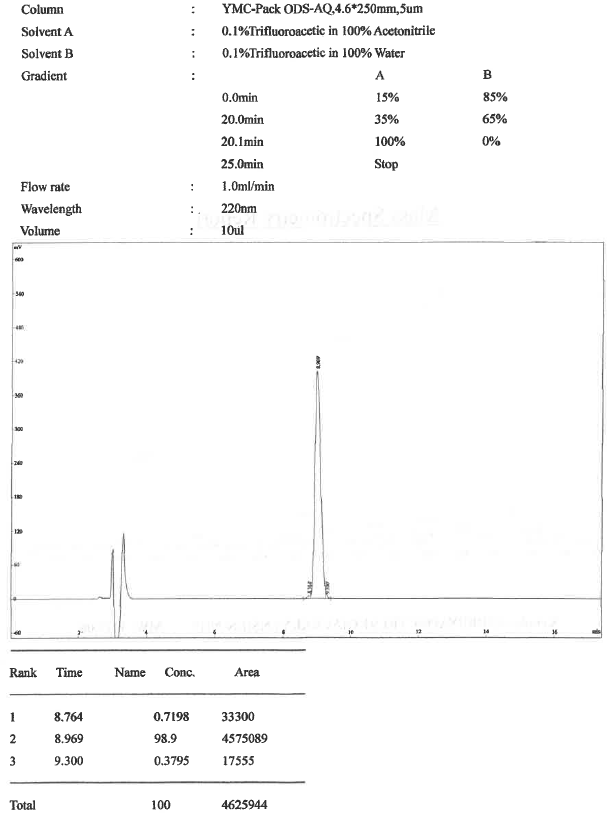

Supplement: Supplementary Material 1 [file mmc1.docx]
